# Phytochemical and Antioxidant Profiling of Traditional Fruit Vinegars: A Comparative Study of Fruit Species

**DOI:** 10.1002/fsn3.71481

**Published:** 2026-02-17

**Authors:** Ayşen Melda Çolak, Fatma Alan, İbrahim Bulduk, Civan Çelik

**Affiliations:** ^1^ Faculty of Agriculture, Department of Horticulture Uşak University Uşak Türkiye; ^2^ Department of Plant and Animal Production, Kalecik Vocational School Ankara University Ankara Türkiye; ^3^ Faculty of Engineering, Department of Chemical Engineering Afyon Kocatepe University Afyonkarahisar Türkiye; ^4^ Agriculture Faculty, Department of Agricultural Biotechnology Isparta University of Applied Science Isparta Türkiye

**Keywords:** bioactive compounds, *citrus aurantium*, fruit vinegar, natural fermentation, phytochemical composition, *rubus fruticosus*, *viburnum opulus*

## Abstract

In this study, natural fruit vinegars produced from gilaburu ( *Viburnum opulus* ), blackberry ( *Rubus fruticosus* ), and bitter orange ( *Citrus aurantium* ) using traditional fermentation methods were compared in terms of their phytochemical composition and antioxidant capacity. Total phenolic content (496.07–696.0 mg GAE·L^−1^), flavonoid levels (376.10–960.97 mg QE·L^−1^), antioxidant activity (DPPH assay, 82.97%–92.02%), and organic acid profiles particularly oxalic acid (105.00–109.33 mg·L^−1^) and acetic acid (288.00–308.67 mg·L^−1^) were analyzed. The results revealed statistically significant differences among vinegar types in terms of total phenolic compounds, flavonoid levels, and certain organic acids (*p* < 0.001). Blackberry and gilaburu vinegars exhibited the highest total phenolic content, whereas the highest flavonoid level was determined in blackberry vinegar. The lowest flavonoid levels were observed in bitter orange vinegar, whereas bitter orange vinegar was distinguished by its acetic acid content within the organic acid profile. Despite having relatively lower phenolic and flavonoid contents, bitter orange vinegar demonstrated a high antioxidant activity comparable to that of the other vinegars (*p* > 0.05). This finding is likely attributable to synergistic effects between organic acids and other bioactive compounds. Phytochemical variation and sample grouping were visualized using principal component analysis, indicating that the observed differences were largely driven by the genetic characteristics of the fruit species because of the study design, ecological effects could not be evaluated independently of genetic factors. Overall, the findings suggest that blackberry and gilaburu vinegars possess a higher potential as functional food products, whereas bitter orange vinegar represents a valuable alternative owing to its strong antioxidant capacity and distinctive organic acid profile.

## Graphical abstract

The graphical abstract illustrates the phytochemical composition and antioxidant capacity of gilaburu (*Viburnum opulus*), blackberry (*Rubus fruticosus*), and bitter orange (*Citrus aurantium*) vinegars, highlighting species‐specific differences in functional properties.

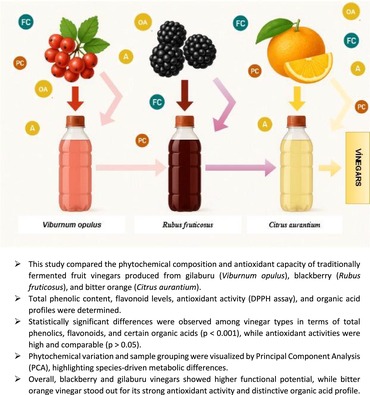

## 1 Introduction

Berry fruits and citrus fruits are among the important plant sources for human health because of their high levels of bioactive compounds. These fruits are rich in various phytochemicals such as total phenolic compounds, flavonoids, ascorbic acid, organic acids, and anthocyanins. Owing to these components, they possess high antioxidant capacities, support the immune system, and may exert protective effects against chronic diseases such as cardiovascular disorders, diabetes, and certain types of cancer (Sen and Chakraborty 2011; Karabulut and Yemiş 2019; Gou et al. 2017).

Among the berry fruit group, blackberry ( *Rubus fruticosus* ) stands out for its rich anthocyanin and flavonol content, whereas gilaburu ( *Viburnum opulus* ), traditionally used in Türkiye's Central Anatolia Region, is known for its strong antioxidant effects (Çolak et al. 2022; Kajszczak et al. 2020). On the other hand, bitter orange ( *Citrus aurantium* ), a citrus fruit species, is notable for its high vitamin C, flavanone, and citric acid content (Hsouna et al. 2023; Sezen et al. 2021).

These bioactive compounds are critically important not only for human health but also for the physiological defense mechanisms of plants. Phenolic compounds and flavonoids are secondary metabolites that play a key role in plant defense against environmental factors such as UV radiation, pathogen attacks, and oxidative stress (Sreekumar et al. 2022). The biosynthesis of these compounds is regulated by the phenylpropanoid pathway, in which enzymes such as phenylalanine ammonia‐lyase (PAL), chalcone synthase (CHS), and flavonol synthase (FLS) are involved (Ahmet et al. 2021). Moreover, genotypic variations among species lead to significant differences in the accumulation of these metabolites, and in this study, potential ecological effects cannot be separated from genetic differences because of the experimental design (Zhao et al. 2023). For instance, species such as gilaburu, blackberry, and bitter orange exhibit diverse phenolic and flavonoid profiles largely because of their genetic makeup. Although these fruits were collected from different regions, the observed differences in phytochemical composition primarily reflect species‐specific genetic traits rather than regional ecological effects (Liu et al. 2023). The high proanthocyanidin levels of gilaburu adapted to cold climates, the rich anthocyanin content of blackberry, and the high flavanone and citric acid levels of bitter orange are typical examples of this biological adaptation (Xing et al. 2022).

These fruits can be consumed not only fresh but also processed into fermented products. Vinegar is a traditional product of health significance, produced through a two‐step fermentation process. In this process, yeasts first convert sugars into ethanol, which is then oxidized to acetic acid by *Acetobacter* species (Nosratabadi et al. 2024; Jin et al. 2025). During this process, a significant portion of the biochemical constituents of the fruits is preserved and may even be transformed into more beneficial forms. Interest in natural, additive‐free fruit vinegars has increased significantly in recent years (Ousaaid et al. 2022; Tanyeri and Arısoy 2023). Particularly, converting nutritionally rich and regionally important fruits such as gilaburu, blackberry, and bitter orange into value‐added fermented products offers a promising solution for sustainable agricultural practices and local producer support mechanisms, contributing to rural development (Gangakhedkar et al. 2025). The use of these fruits in vinegar production is important not only for the processing industry but also for the conservation of agricultural biodiversity, the promotion of traditional fruit cultivation, and rural development (İlter 2019). However, comparative scientific data on the phytochemical composition and health‐promoting properties of vinegars produced from these botanically distinct fruits remain limited (Bozdemir et al. 2020). In this context, evaluating how ecological conditions and species‐specific genetic differences are reflected in the phytochemical composition of fermented products such as vinegar is of great importance for elucidating the relationship between plant metabolism, environmental adaptation, and functional food quality. However, it was clearly stated that the phytochemical differences observed in the study were largely attributable to the genetic makeup of the species and that ecological conditions could not be evaluated independently of genetic effects.

The aim of this study was to comparatively evaluate the total phenolic content (TPC), flavonoid levels, DPPH antioxidant activities, and organic acid profiles of natural vinegars produced from gilaburu, blackberry, and bitter orange fruits using traditional fermentation methods, and to investigate the potential effects of regional ecological conditions and fruit genotypic differences on the accumulation of these bioactive compounds. In this context, the study aims to reveal the potential health benefits of different fruit vinegars for human health, to promote the utilization of local fruit varieties, and to provide a scientific basis for future sustainable product development processes.

## 2 Materials and Methods

### 2.1 Plant Material

The fruit samples used in this study were collected during the 2023 harvest season from naturally growing plants at their commercial maturity stage. Bitter orange ( *Citrus aurantium* ) fruits were collected from the Seferihisar district of Aydın Province in the Aegean Region of Türkiye, whereas blackberry ( *Rubus fruticosus* ) and gilaburu ( *Viburnum opulus* ) fruits were obtained from the Kululu and Akbayır areas of the Akkışla district, Kayseri Province, located in the Central Anatolia Region (Table 1). The collected fruits represent common species that grow spontaneously in their natural habitats and no specific cultivar or genotype identification was performed.

**TABLE 1:** Genotypes of fruit vinegars used in the study and their collection locations.

Genotypes	Location	Altitude (m)	Coordinates	
			Latitude	Longitude
Gilaburu	Kayseri‐Akkışla‐Kululu‐Kale	1423	38° 58′ 33″ N	36° 08′ 2 0″ E
Blackberry	Kayseri‐Akkışla‐Akbayır	1370	39° 00′ 08″ N	36°10′25″ E
Bitter orange	Aydın‐Buharkent‐Menderes	164	37° 57′ 36″ N	28°44′26″ E

Prior to production, all fruits were washed under running water to remove surface dirt, dust, and foreign materials. Vinegar production was conducted in triplicate for each fruit type, with 5.0 kg of fresh fruit used per replicate. Bitter orange fruits were sliced with their peels into approximately 1 cm thick pieces, whereas blackberry and gilaburu fruits were crushed and placed whole into the fermentation vessels. Each fruit sample was mixed with natural honey at a ratio of 15%–20% (w/w), depending on the fruit quantity, and approximately 1 L of chlorine‐free drinking water was added to ensure complete submersion of the fruits. Each replicate was prepared in approximately 10 L glass fermentation containers. The openings of the fermentation containers were covered and secured with cheesecloth to allow air exchange while preventing the entry of dust and microorganisms from the external environment. Fermentation was carried out for 3–4 weeks at a temperature range of 18°C–25°C in a dark and cool environment. During this period, the mixtures were gently stirred every 2 to 3 days to ensure homogeneous distribution and adequate oxygen circulation. After completion of fermentation, the fruit pulp was filtered out, and the resulting liquids were transferred into clean glass bottles. The bottles were stored in a cool and dark environment for 1–2 months to allow vinegar maturation. At the end of this process, bitter orange, blackberry, and gilaburu vinegars were prepared independently for each replicate for subsequent chemical analyses.

### 2.2 Chemical Characteristics

The total phenolic compounds, flavonoids, and organic acid contents were analyzed in natural vinegar samples produced from gilaburu, blackberry, and bitter orange fruits. In this context, the analytical methods used to determine the phytochemical properties of the vinegar samples are detailed below.

#### 2.2.1 Determination of TPC

The TPC in fruit juice samples was measured using the Folin–Ciocalteu method with slight modifications (Gámez‐Meza et al. 1999). Prior to analysis, samples were filtered through a 0.45 μm membrane filter. Gallic acid, a known phenolic acid, was used to prepare calibration standards at concentrations of 500, 250, 100, 80, 60, 40, 20, and 10 ppm in methanol. Absorbance was recorded at 765 nm, with standard concentrations plotted on the *x*‐axis and absorbance on the *y*‐axis to generate a calibration curve. The curve exhibited a high linearity with an *R* ^2^ value of 0.9997, demonstrating the method's accuracy and precision. Using this calibration, TPCs of the samples were calculated and expressed as mg GAE·L^−1^ dry matter. For the assay, 0.1 mL of sample was mixed with 7.9 mL distilled water, 0.5 mL Folin–Ciocalteu reagent, and sodium carbonate solution (20%), then incubated at 40°C for 30 min. Absorbance was subsequently measured at 765 nm using a UV–Vis spectrophotometer. The same procedure was applied to gallic acid standards, and methanol was used as the blank.

#### 2.2.2 Determination of Total Flavonoid Content (TFC)

TFC in fruit juice samples was determined by the aluminum chloride colorimetric method developed by Woisky and Salatino (1998). Samples were diluted with distilled water to 500 ppm. Quercetin (QE) standards were prepared in methanol at concentrations of 500, 400, 300, 200, 100, 50, and 25 ppm to construct the calibration curve. Absorbance readings at 415 nm were plotted against standard concentrations to obtain the calibration curve with an *R* ^2^ of 0.9996, indicating high sensitivity. For analysis, 0.5 mL of 500 mg/L sample was mixed with 1.5 mL methanol, 0.1 mL AlCl_3_ solution, and 0.1 mL 1 M sodium acetate, then incubated at room temperature for 30 min. Absorbance was measured at 415 nm. The same procedure was followed for quercetin standards, with methanol used as the blank.

#### 2.2.3 Antioxidant Activity (DPPH)

Antioxidant activity was measured using the DPPH (2,2‐diphenyl‐1‐picrylhydrazyl) radical scavenging assay, with minor modifications to the established protocol (Ebrahimzadeh et al. 2008). The stock DPPH solution (6 × 10^−5^ M) was prepared by dissolving 0.0024 g of DPPH in 100 mL of methanol. Working DPPH solution (40 mg L^−1^) was obtained by diluting the stock with methanol. For the assay, 300 μL of sample extract was mixed with 5700 μL of the working DPPH solution in a 10 mL test tube. The mixture was thoroughly vortexed and incubated in the dark at room temperature for 60 min. After incubation, absorbance was measured at 517 nm using a Shimadzu UV‐1800 UV–Vis spectrophotometer. A control containing no extract was prepared and measured under the same conditions. Antioxidant activity (%) was calculated using the formula:

$$\text{Antioxidant Activity}\;\left(\%\right)=\left[\left(\mathrm{AC}{\left(O\right)}_{517}-\mathrm{AA}{\left(t\right)}_{517}\right)/\mathrm{AC}{\left(O\right)}_{517}\right]\times 100$$

Here, AC (O)_517_ represents the absorbance of the control at time zero (*t* = 0 min) and AA (*t*)_517_ represents the absorbance of the sample after 1 h (*t* = 1 h).

### 2.3 Statistical Analysis

All statistical analyses were performed using IBM SPSS Statistics 21.0 software (IBM Corp 2013). Descriptive statistics (mean, standard deviation, coefficient of variation, etc.) were calculated for the obtained phytochemical data. One‐way analysis of variance (ANOVA) was applied to determine differences between groups, with a significance level set at *p* < 0.05. Following ANOVA, Tukey's Multiple Comparison test was used for pairwise comparisons between groups. Additionally, principal component analysis (PCA) was conducted to identify relationships among variables and the main sources of variation. PCA results were supported by the explained variance percentages and eigenvalue values of the components.

## 3 Results

### 3.1 Phytochemical Profile and Antioxidant Properties of Fruit Vinegars

The data presented in Table 2 clearly reveal the differences in phytochemical contents and antioxidant capacities among gilaburu, blackberry, and bitter orange vinegars.

**TABLE 2:** Phytochemical profile and antioxidant properties of fruit vinegars (Mean ± SD; CV).

	Abbreviation	Unit	Gilaburu	Blackberry	Bitter orange	CV (%)
Total phenolic content	TPC	mg GAE·L^−1^	695.77 ± 0.87 a	696.00 ± 0.80 a	497.07 ± 0.80 b	15.79
Total flavonoid content	TFC	mg QE/L	624.17 ± 0.67 b	960.97 ± 0.25 a	376.10 ± 0.95 c	38.89
Antioxidant activity						
Antioxidant activity (DPPH)	AntAc	%	82.97 ± 17.55 a	90.00 ± 0.40 a	92.03 ± 0.55 a	10.98
Organic acids						
Oxalic acid	OxaA	mg·L^−1^	105.00 ± 3.00 c	183.00 ± 2.65 a	109.33 ± 4.16 b	28.75
Tartaric acid	TarA	mg·L^−1^	196.33 ± 10.60 a	202.33 ± 6.35 a	207.67 ± 4.73 a	4.08
Acetic acid	AceA	mg·L^−1^	299.33 ± 3.06 b	308.67 ± 4.73 a	288.00 ± 4.00 c	3.22
Citric acid	CitA	mg·L^−1^	2.00 ± 0.00 a	1.00 ± 0.00 b	1.00 ± 0.00 c	37.50

*Note:* Values with different superscript letters (a–c) within a row are significantly different at *p* < 0.05 (Tukey’s HSD test).

Abbreviations: CV, coefficient of variation; SD, standard deviation.

In terms of TPC, gilaburu (695.77 mg GAE·L^−1^) and blackberry (696.00 mg GAE·L^−1^) showed similar and high values, whereas bitter orange (497.07 mg GAE·L^−1^) exhibited significantly lower phenolic content. This indicates that gilauru and blackberry vinegars possess a richer phenolic compound profile and thus potentially offer higher antioxidant capacity. The coefficient of variation (CV) was 15.79%, indicating moderate variability.

Regarding TFC, blackberry (960.97 mg QE/L) had the highest value, followed by gilaburu (624.17 mg QE/L). Bitter orange (376.10 mg QE/L) stood out with a considerably lower flavonoid content. The high variation coefficient in flavonoids (38.89%) suggests substantial differences among samples.

Although antioxidant activity (DPPH) values were high in all three types, gilaburu (82.97%) was somewhat lower compared to the others. Blackberry (90.00%) and bitter orange (92.03%) vinegars exhibited similar and high antioxidant activities. The CV value of 10.98% indicates relatively consistent antioxidant capacities.

When considering organic acids, oxalic acid content was highest in blackberry vinegar (183.00 mg·L^−1^), whereas gilaburu (105.00) and bitter orange (109.33 mg·L^−1^) showed lower amounts. The high variation in oxalic acid content (28.75%) points to significant differences among the species. Tartaric acid content was similar and high across all samples, with no significant differences observed among gilaburu, blackberry, and bitter orange vinegars (CV 4.08%).

Acetic acid content was close between blackberry (308.67 mg·L^−1^) and gilaburu (299.33 mg·L^−1^), whereas bitter orange (288.00 mg·L^−1^) had slightly lower levels. These values suggest a possible contribution to the vinegar's taste and aroma characteristics. The low variation in acetic acid content (CV 3.22%) reflects consistency among samples. Citric acid was highest in gilaburu vinegar (2.00 mg·L^−1^), with lower amounts in blackberry and bitter orange (1.00 mg·L^−1^). However, the low absolute values resulted in a high CV (37.50%). These differences in citric acid levels were observed to play a significant role in taste and acidity.

Overall, gilaburu and blackberry vinegars were observed to be richer in phenolic and flavonoid components, highlighting species‐specific differences rather than ecological influences. Although bitter orange vinegar had lower phenolic and flavonoid contents compared to the others, it exhibited high antioxidant activity, likely because of the synergistic effects of different compounds, emphasizing interspecies variation.

### 3.2 One‐Way ANOVA Results for Phytochemical Parameters

A one‐way ANOVA test was conducted to evaluate group differences among the phytochemical parameters (Table 3).

**TABLE 3:** ANOVA results for phytochemical parameters of fruit vinegars.

	Sum of squares	Mean square	*F*	Sig.
Total phenolic content (TPC)				
Between groups	79056.21	39528.10	57940.22	0.001
Within groups	4.093	0.682		
Total	79060.30			
Total flavonoid content (TFC)				
Between groups	517040.32	258520.16	547454.46	0.001
Within groups	2.833	0.472		
Total	517043.16			
Antioxidant activity				
Antioxidant activity (DPPH) (AntAc)				
Between groups	135.80	67.903	0.66	0.55
Within groups	617.05	102.84		
Total	752.86			
Organic acids				
Oxalic acid (OxaA)				
Between groups	11529.55	5764.78	518.83	0.001
Within groups	66.66	11.11		
Total	11596.22			
Tartaric acid (TarA)				
Between groups	192.88	96.44	1.65	0.26
Within groups	350.00	58.33		
Total	542.88			
Acetic acid (AceA)				
Between groups	642.66	321.33	20.22	0.001
Within groups	95.33	15.88		
Total	738.00			
Citric acid (CitA)				
Between groups	2.00	1.00		
Within groups	0.00	0.00		
Total	2.00			

Because of the extremely low within‐group variance for TPC and TFC, very high F‐values were observed. This indicates that the measurements exhibited high repeatability and that experimental error was minimal, which in turn led to a more pronounced statistical differentiation among groups. Therefore, the extremely high F‐values reported in Table 3 (e.g., 57,940.22) should be regarded as statistically reliable and methodologically expected results. A significant difference was found between groups for TPC. Similarly, a statistically significant difference was observed between groups for TFC. However, no significant difference was detected among groups for antioxidant activity (DPPH), indicating that the samples had comparable antioxidant capacities. Regarding organic acids, highly significant differences were found between groups for oxalic acid (OxaA) content. Significant differences were also observed for acetic acid (AceA), whereas tartaric acid (TarA) did not show significant variation among groups. Because of insufficient within‐group variance in the citric acid (CitA) data, a meaningful variance analysis could not be performed.

These results indicate that TPC, TFC, oxalic acid, and acetic acid levels differed significantly among the examined fruit vinegar samples, whereas no significant differences were observed in antioxidant activity, tartaric acid, and citric acid contents.

### 3.3 Multiple Comparison (Tukey) Analysis of Phytochemical Parameters

Multiple comparison analyses revealed significant differences in phytochemical component levels among different fruit vinegar groups (Table 4).

**TABLE 4:** Tukey multiple comparison results for phytochemical parameters of fruit vinegars.

						95% confidence Interval	
	(I) Sample	(J) Sample	Mean difference (I–J)	Std. error	Sig.	Lower bound	Upper bound
TPC	Gilaburu	Blackberry	−0.23	0.67	0.93	−2.30	1.84
	Gilaburu	Bitter orange	198.70*	0.67	< 0.001	196.63	200.77
	Blackberry	Bitter orange	198.93*	0.67	< 0.001	196.86	201.00
TFC	Gilaburu	Blackberry	−336.80*	0.56	< 0.001	−338.52	−335.08
	Gilaburu	Bitter orange	248.07*	0.56	< 0.001	246.35	249.79
	Blackberry	Bitter orange	584.87*	0.56	< 0.001	583.15	586.59
Antioxidant activity							
DPPH (%)	Gilaburu	Blackberry	−7.03	8.28	0.69	−32.44	18.37
	Gilaburu	Bitter orange	−9.07	8.28	0.55	−34.47	16.34
	Blackberry	Bitter orange	−2.03	8.28	0.97	−27.44	23.37
Organic acids							
OxaA	Gilaburu	Blackberry	−78.00*	2.72	< 0.001	−86.35	−69.65
	Blackberry	Bitter orange	73.67*	2.72	< 0.001	65.32	82.02
TarA	Gilaburu	Blackberry	−6.00	6.24	0.63	−25.13	13.13
	Gilaburu	Bitter orange	−11.33	6.24	0.24	−30.47	7.80
	Blackberry	Bitter orange	−5.33	6.24	0.69	−24.47	13.80
AceA	Gilaburu	Blackberry	−9.33	3.26	0.06	−19.32	0.65
	Gilaburu	Bitter orange	11.33*	3.26	0.03	1.35	21.32
	Blackberry	Bitter orange	20.67*	3.26	0.002	10.68	30.65

* The mean difference is significant at the 0.05 level.

Regarding TPC, bitter orange samples were found to have significantly higher values compared to both gilaburu and blackberry samples (*p* < 0.001). No statistically significant difference was observed between gilaburu and blackberry (*p* = 0.93). In terms of TFC, blackberry samples exhibited significantly lower flavonoid levels than gilaburu (*p* < 0.001), whereas bitter orange samples differed significantly from both groups (*p* < 0.001). These results indicate that blackberry had the lowest and bitter orange the highest flavonoid content. No statistically significant differences were found between groups for antioxidant activity (DPPH) (*p* > 0.05), indicating that the vinegar samples generally had similar antioxidant capacities. Looking at the organic acid profile, significant differences in oxalic acid (OxaA) levels were observed between gilaburu and blackberry and between blackberry and bitter orange (*p* < 0.001). However, no significant difference was detected between gilaburu and bitter orange (*p* = 0.31). Additionally, tartaric acid (TarA) contents were similar among groups, with no significant differences (*p* > 0.05). For acetic acid (AceA) levels, bitter orange showed significantly higher acetic acid content compared to both gilaburu (*p* = 0.030) and blackberry (*p* = 0.002). The difference between gilaburu and blackberry was borderline but not statistically significant (*p* = 0.064). Variance analysis could not be performed for citric acid (CitA), or no significant differences were found between groups; this may be due to similar values measured across all samples or insufficient variance for this parameter (Table 4).

These findings demonstrate significant phytochemical differences among the fruit vinegars, particularly in terms of phenolic and flavonoid compounds as well as certain organic acids; such differences may influence the functional properties and potential health benefits of the products.

### 3.4 Principal Component Analysis (PCA)

The results of PCA, conducted to identify the underlying structure among variables in the dataset and for dimensionality reduction, are presented in Table 5.

**TABLE 5:** Explained variance and eigenvalue results from principal component analysis (PCA) of phytochemical parameters of fruit vinegars.

Component	Initial eigenvalues			Extraction sums of squared loadings		
	Total	% of variance	Cumulative %	Total	% of variance	Cumulative %
PC1	3.46	49.47	49.47	3.46	49.47	49.47
PC2	2.05	29.29	78.76	2.05	29.29	78.76
PC3	1.21	17.33	96.09	1.21	17.33	96.09
PC4	0.19	2.78	98.88			
PC5	0.08	1.10	99.98			
PC6	0.00	0.02	100.00			
PC7	0.00	0.00	100.00			

The first principal component (PC1) explained 49.47% of the total variance, indicating that nearly half of the variability in the dataset could be represented by a single component. The second component (PC2) accounted for 29.29% of the variance, whereas the third component (PC3) explained 17.33%, resulting in a cumulative variance of 96.09% for the first three components. This high cumulative percentage indicates that the extracted components are sufficient to represent the majority of the dataset. Since the eigenvalues for the fourth component (PC4) and subsequent components were below 1 and their explained variance ratios were less than 3%, they were excluded from further statistical analyses. Therefore, for a better understanding of the data structure, it is recommended to use PC1, PC2, and PC3 in subsequent analyses (e.g., clustering or correlation analysis).

**FIGURE 1:**
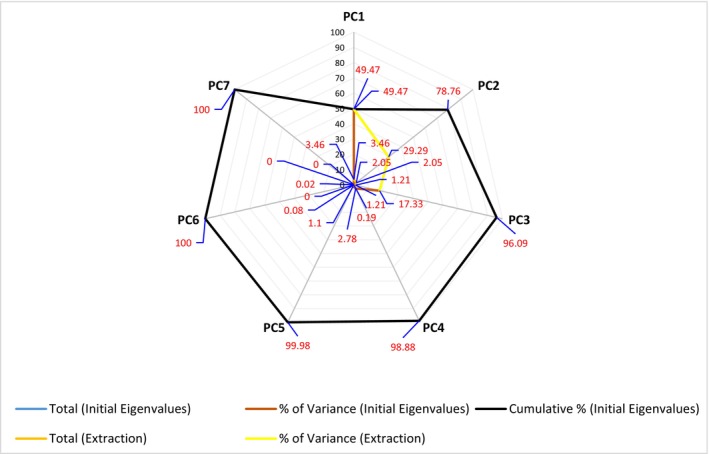
PCA scree plot showing the distribution and clustering of blackberry, gilaburu, and bitter orange vinegar samples on the basis of phytochemical parameters (PC1 vs. PC2).

Figure 1 shows the clustering of blackberry, gilaburu, and bitter orange vinegar samples on the basis of PC1 and PC2. Each sample was evaluated in triplicate. A total of seven components (PC1–PC7) were obtained from the PCA, with the first three components explaining approximately 96% of the total variance, effectively representing the main variation in the dataset.

In the scatter plot, samples of the same type clustered closely together, reflecting similar phytochemical profiles, whereas different sample types were clearly separated, indicating distinct compositional differences. Because of the limited number of samples, the labels on the graph were highly readable, allowing meaningful differences to be clearly observed in the analysis. These results suggest that the variables measured in this study were effective in explaining the variation among samples and confirm that PCA is a suitable method for reducing dimensionality and visualizing relationships among the vinegar samples.

## 4 Discussion

In this study, significant differences were observed among the three analyzed fruit vinegars (gilaburu, blackberry, and bitter orange) primarily because of species‐specific phytochemical profiles rather than direct ecological effects. References to ecological adaptation are retained as literature‐based context but are not drawn as conclusions from the current data. These differences are thought to result not only from interspecific botanical distinctions but also from the interaction of genetic and ecological factors. However, it should be emphasized that the present study is based on compositional analyses and does not include molecular or gene expression data. These data demonstrate that the compositional differences among fruit vinegars primarily reflect species‐specific genetic traits, thereby influencing both their bioactive potential for human health and their sensory properties.

The differences observed in phenolic accumulation in this study reflect interspecies variation among the examined vinegars. In the literature, it has been reported that enzymes such as phenylalanine ammonia‐lyase (PAL) and chalcone synthase (CHS) play a role in phenolic biosynthesis in response to environmental stress conditions, such as UV radiation and high temperature. However, these mechanisms are presented solely for contextual explanation and should not be interpreted as direct findings of the present study (Jie et al. 2023; Song et al. 2022).

Similarly, in berry fruits such as blackberry and gilaburu, earlier studies have reported that variations in altitude‐related temperature and light intensity can influence the expression of genes regulating flavonoid biosynthesis enzymes, leading to changes in anthocyanin and flavonol accumulation (Rieger et al. 2008; Dossett et al. 2012). These observations are cited here solely to contextualize the current findings, as no direct assessment of gene expression or enzymatic activity was performed in this study.

Furthermore, the pronounced effect of ecological region differences on phytochemical profiles was also supported by PCA clustering analyses. The gilaburu samples, originating from Central Anatolia and adapted to relatively continental climatic conditions, showed a distinct separation in terms of TPC, whereas the bitter orange samples, adapted to Mediterranean climatic conditions, exhibited higher flavanone‐related characteristics. Such clustering patterns suggest that ecological adaptation may influence phytochemical composition, as previously highlighted in studies emphasizing the combined effects of genetic background and environmental conditions on secondary metabolite diversity (Yi et al. 2014).

Bitter orange vinegar exhibited the lowest TPC and TFC values compared to the other two vinegars, which contrasts with the findings of Yi et al. (2014), who reported high phenolic and flavonoid levels in immature *Citrus unshiu* vinegars. This discrepancy may be attributed to differences in species, fruit maturity stage, processing conditions, and fermentation dynamics rather than direct molecular regulation (Yi et al. 2014). Although phenylpropanoid metabolism has been reported to be highly active in *Citrus* species, any reference to enhanced PAL activity in bitter orange should be regarded as a literature‐based hypothesis rather than a conclusion derived from the present data (Lado et al. 2018).

Overall, the findings indicate that both genetic control and environmental regulation should be considered together in the biosynthesis of secondary metabolites. Future studies incorporating molecular approaches, such as the analysis of PAL, CHS, and flavonol synthase (FLS) gene expression under different environmental stress conditions, would be required to verify these hypotheses and clarify the underlying mechanisms (Jie et al. 2023; Dossett et al. 2012; Yi et al. 2014).

Blackberry vinegar exhibited relatively low TPC and TFC values in the present study. However, Boonsupa (2020) and Güzel (2024) reported higher phenolic contents in blackberry vinegars. This discrepancy may be attributed to several interrelated factors. First, the genotype used in our study ( *Rubus idaeus* ) differs from that in previous studies ( *Rubus fruticosus* ), and genotypic variation has been widely reported to influence phenolic accumulation. Second, differences in cultivation conditions such as soil composition, climate, and altitude may indirectly affect secondary metabolite synthesis through plant stress responses. Third, fermentation duration and microbial activity may alter phenolic stability and extraction efficiency (Boonsupa 2020; Güzel 2024). Additionally, as reported by Pădureanu et al. (2022), phenolic compounds in berry fruit vinegars may be inversely correlated with acetic acid content, which could partially explain the relatively lower TPC values observed in blackberry vinegar in this study. These factors highlight that phenolic and flavonoid contents alone may not fully reflect the functional complexity of vinegar products (Pădureanu et al. 2022).

Gilaburu vinegar exhibited moderate levels of phenolic and flavonoid content; however, previous studies have emphasized that gilaburu vinegar is notable not only for its phenolic profile but also for its free amino acids, mineral composition, and potential health‐related properties (Erdal et al. 2022). In the present study, the organic acid profile of gilaburu vinegar revealed relatively higher levels of citric and malic acids compared to the other vinegars, which may contribute to its functional attributes. These findings suggest that gilaburu vinegar possesses a multifunctional phytochemical profile, despite its moderate phenolic content.

In terms of antioxidant activity, DPPH analysis revealed no statistically significant differences among the samples, and all vinegars exhibited high antioxidant capacity. Previous studies have emphasized that fermentation conditions play a decisive role in antioxidant activity. Although the vinegars differed significantly in total phenolic and flavonoid contents, these differences did not translate into detectable changes in DPPH activity. This may be due to the contribution of multiple antioxidant compounds, including organic acids and other non‐phenolic constituents, as well as the limited sensitivity of the DPPH assay in complex matrices, which has been reported to have constraints in discriminating antioxidant systems with different reaction kinetics and synergistic interactions (Huang et al. 2005; Prior et al. 2005). Therefore, the antioxidant capacity observed in all samples likely reflects the cumulative effect of diverse bioactive compounds rather than phenolics alone. In terms of antioxidant activity, values measured by the DPPH method showed no statistically significant differences among the samples, and all vinegars exhibited high antioxidant capacity. Chen et al. (2016) and Karaağaç and Gün (2024) emphasized that fermentation conditions play a decisive role in antioxidant activity. The similar antioxidant levels observed in the present study suggest that the fermentation and production parameters were closely aligned across all vinegar samples, thereby minimizing variability in antioxidant performance despite differences in phytochemical composition (Chen et al. 2016; Karaağaç and Gün 2024).

When examining the organic acid profile, bitter orange vinegar stood out with its high acetic acid content. Although Cunha et al. (2016) reported acetic acid productivity in blackberry vinegars, the present findings indicate that citrus species such as bitter orange may possess a greater capacity for acetic acid production. This may be attributed to substrate characteristics in bitter orange juice, including its sugar composition and organic acid precursors, which support robust microbial activity during fermentation (Cunha et al. 2016).

Regarding organic acids, bitter orange vinegar exhibited the highest acetic acid content. This observation may be related to the high sugar content of bitter orange juice, which supports robust microbial activity during fermentation. Overall, the phytochemical variability among gilaburu, blackberry, and bitter orange vinegars appears to be shaped by a combination of genetic background, ecological adaptation, and processing conditions. Although phenolic compounds and flavonoids are known to play important roles in plant stress tolerance, their regulation at the molecular level was not investigated in this study and should be addressed in future research.

## 5 Conclusion

This study revealed interspecific phytochemical differences among vinegars produced from gilaburu, blackberry, and bitter orange fruits, highlighting in particular the functional potential of blackberry and gilaburu vinegars. Phytochemical analyses demonstrated that significant differences in TPC, flavonoid levels, and certain organic acids were attributable solely to the species‐specific genetic characteristics of the plant materials. Ecological effects were not included within the scope of the study, and information from the literature was used only in a discussion context; therefore, the observed differences were interpreted as reflecting interspecific variation independent of ecological conditions. These findings underscore the importance of genotype selection in determining the biological activity potential of fruits used in vinegar production and suggest that the evaluation of local genotypes through sustainable agricultural practices may contribute to both functional product development and the conservation of biological diversity. Future studies incorporating molecular approaches may enable a more detailed investigation of interspecific metabolite accumulation and functional properties.

## Author Contributions

F.A. conceptualized the study, planned and coordinated the experimental design and was responsible for the collection of all samples. She performed all statistical analyses, interpreted the data, prepared the results and contributed to the discussion of the statistical findings. F.A. also took charge of writing and editing the manuscript and supervised all aspects of the study. A.M.Ç. conceptualized the study, planned and coordinated the experimental design, contributed to the writing and editing of the manuscript, conducted the sample analyses in the laboratory and assisted in all aspects of the study. İ.B. and C.Ç. actively contributed to the laboratory work and sample analyses, assisted in data interpretation, and provided scientific input in the execution of the study. Both also contributed to the discussion of the findings and reviewed the manuscript.

## Funding

The authors have nothing to report.

## Ethics Statement

The authors have nothing to report.

## Consent

The authors have nothing to report.

## Conflicts of Interest

The authors declare no conflicts of interest.

## Data Availability

The data that support the findings of this study are available from the co‐corresponding authors upon reasonable request.
